# Biochemical changes in tuberculosis

**DOI:** 10.11604/pamj.2021.38.66.21707

**Published:** 2021-01-20

**Authors:** Chinyelu Uchenna Ufoaroh, Chidimma Adaobi Onwurah, Victor Ahaoma Mbanuzuru, Chidimma Ifeyinwa Mmaju, Shirley Nneka Chukwurah, Chukwudi Christian Umenzekwe, Iloduba Nnaemeka Aghanya, Simon Nkpeh Ushie, Arthur Ebelenna Anyabolu, Emeka Hyacinth Enemuo, Ernest Ndukaife Anyabolu, Prince Udegbunam Ele

**Affiliations:** 1 Department of Internal Medicine, Nnamdi Azikiwe University, Anambra State, Nigeria,; 2 Department of Community Medicine, Nnamdi Azikiwe University Teaching Hospital, Nnewi, Nigeria,; 3 Department of Physiology, Nnamdi Azikiwe University, Anambra State, Nigeria,; 4 Department of Medical Microbiology and Parasitology, Nnamdi Azikiwe University, Anambra State, Nigeria,; 5 Department of Internal Medicine, Chukwuemeka Odumegwu Ojukwu University, Awka, Nigeria

**Keywords:** Tuberculosis, serum calcium, serum albumin, serum sodium, serum potassium, Southeast Nigeria

## Abstract

**Introduction:**

tuberculosis (TB) remains a global health issue with high morbidity and mortality rates especially in the developing countries. It is a multi-organ disease and can influence biochemical changes. This study sought to determine the influence of tuberculosis and its drug treatment on serum biochemical parameters in patients in Nigeria.

**Methods:**

it was a descriptive observational cohort study on 150 subjects whose blood samples were analyzed for serum albumin, serum sodium, and serum potassium. The subjects were grouped into 3: TB group= 50 new TB subjects not on treatment, F group= 50 TB subjects on treatment for 2/12 or more and C group= 50 non-TB control subjects. These biochemical variables were compared between the 3 groups.

**Results:**

male/female ratio was 1: 1.5, mean age 37.1±0.92 years, and range 18-65 years. The differences in mean values of serum albumin, calcium and sodium between the three groups were significant (p<0.001), whereas that of serum potassium was not significant (p=0.056). Those patients with new case TB had a significantly lower serum sodium, serum albumin and serum calcium than the control group and those on treatment, p<0.001. There was significant positive correlation between serum albumin and serum calcium (r=0.0.420, p<0.001) as well as serum sodium (r=0.310, p<0.001) in the study population. Similarly, the correlation between serum calcium and serum sodium was positive and significant (r=0.200, p=0.014). In contrast, the correlation between serum potassium and serum albumin and that between serum potassium and serum calcium was not significant.

**Conclusion:**

tuberculosis with or without anti-tuberculous medications was associated with significant reduction in serum albumin, serum sodium and serum calcium in this study.

## Introduction

Tuberculosis remains a global health issue with high morbidity and mortality rates especially in developing countries with estimated new cases of 10 million annually and with a total of 1600 deaths annually [1]. Tuberculosis is caused by the *Mycobacterium tuberculosis* complex. It is treatable and preventable. Several factors like human immunodeficiency virus (HIV) co-infection and other co-morbidities may affect outcome if not properly addressed. High TB prevalence, according to global view, is reported in sub-Saharan Africa, Asia and India [2]. These are regions with high rates of poverty and poor living conditions [2].

Studies have shown that TB was associated with biochemical changes [3], namely low serum sodium, low serum albumin, low/high serum calcium, low/high serum potassium, among others [4-8]. Furthermore, two studies have demonstrated some biochemical changes associated with anti-TB agents [9, 10]. There is a paucity of studies on the biochemical changes due to TB infection in sub-Saharan African countries, including Nigeria. This prompted this study which sought to evaluate the influence of TB infection and its anti-TB medications on serum albumin, calcium, sodium and potassium, with a view to initiating measures to stem down adverse outcomes from these.

## Methods

**Study design and site:** this was a hospital-based descriptive observational cohort study involving 150 subjects, conducted in Nnamdi Azikiwe University Teaching Hospital, Nnewi, Nigeria, between 2018 and 2019. The study subjects were grouped into 3: TB group= 50 smear positive patients who were not yet on anti-TB drugs, F group= 50 TB patients who had completed 2 months of anti-TB treatment and C group= 50 non-TB subjects as the control group. Pretested questionnaire was administered to each of the study participants. Addressed in the questionnaire were demographic and anthropometric data.

**Exclusion criteria:** participants with established kidney and liver disease, elderly patients, HIV patients, pregnant and lactating patients and children were excluded from the study.

**Consent and approval:** informed written consent was obtained from each of the study participants. The Ethics Research Committee of the hospital gave approval for this study.

**Sample collection:** five (5) ml of whole blood and 20mls of urine were collected from each participant. Urinalysis was carried out on the urine samples while on the blood samples serum albumin, serum sodium, serum potassium, serum calcium were performed. All the tests were conducted in the laboratory of the hospital. The serum biochemical variables were analyzed and compared between the 3 groups in the study population. The association of the serum biochemical variables in the 3 groups was determined.

**Statistical analysis:** this was done with SPSS version 23. Distributive statistics were used for means and frequencies. Correlation statistics were used to determine association between numerical parameters employing pearson´s correlation test. ANOVA was used for inter-group comparisons of mean of the serum biochemical variables, while post hoc test using Bonferroni was employed to determine intra group association. Bivariate post hoc was also used to determine predictors. P<0.05 was considered statistically significant.

**Ethical approval:** this was obtained from NAUTH Ethical Research Committee with reference no NAUTH/CS/66/VOL8/106. Informed consent was also obtained before blood and urine samples were collected.

## Results

The study evaluated 150 subjects comprised of 50 in TB group (smear positive new cases not on treatment), 50 in F-groups (patients that have completed 2 months of intensive treatment from 2 months after initiation of treatment) and 50 in C-group (non-TB control) subjects. There was a female/male ratio of 1.5:1. Female participants were 39.3% while males made up 60.7% of the population. Their overall mean age was 37.11±13.05 years (females 36.08±11.48 years, males 37.78±13.98 years), and age range 18-65 years.

Among the three groups, the mean serum sodium levels were all within the normal values. However, it was high in C group, (non-TB control) (141.50±2.58mmol/l) compared to the F group, (Rx intensive phase) (138.52±3.41mmol/l) as well as TB group, (not on Rx) (136.6±4.12 mmol/l) (Table 1). The differences in the mean values of the serum sodium among these three groups were statistically significant, p<0.001, d=2, mean square=147 (within group), mean square=298.047 (between groups), F=25.264. Furthermore, post-hoc analysis showed a significant difference in the mean values of serum sodium levels in those in group C (non-TB control) compared to F (those Rx >2/12) (p<0.001), TB group (not on Rx) compared to F group (Rx>2/12) (p<0.001), and TB group (not on Rx) compared to C group (non-TB control) (p=0.023) (Table 2).

**Table 1:** mean values of variables in the study population

		Mean + SD	SE	95% CI (Lower-upper) limit	Minimum	Maximum
**Sodium (mmol/l)**	TB group (not on Rx). n=50	136.66±4.129	0.584	135.49 – 137.83	125	145
	Control (non TB). n=50	141.50 ± 2.589	0.366	140.76 – 142.24	136	147
	F (TB ^2^/¹^2^ Rx). n=50	138.52 ± 3.412	0.483	137.55 -139.49	130	145
**Potassium (mmol/l)**	TB group (not on Rx). n=50	4.604 ± 0.937	0.133	4.338 – 4.871	2.51	6.84
	Control (non TB). n=50	4.413 ± 0.916	0.129	4.153 – 4.674	2.42	6.70
	F (TB ^2^/¹^2^ Rx). n=50	4.163 ± 0.886	0.125	3.911 – 4.414	2.38	6.84
**Albumin (g/dl)**	TB (group not on Rx). n=50	31.512 ± 4.34	0.614	30.278 – 32.747	22.45	39.20
	Control (non TB). n=50	38.31 ± 2.02	0.285	37.738 – 38.883	34.71	44.58
	F (TB ^2^/¹^2^ Rx). n=50	36.77 ± 3.86	0.545	35.678 – 37.870	27.17	42.37
**Calcium (mmol/l)**	TB group (not on Rx). n=50	2.17 ± 0.29	0.041	2.085 – 2.252	1.59	3.11
	Control (non TB). n=50	2.35 ± 0.20	0.284	2.294 – 2.408	2.02	2.91
	F (TB ^2^/¹^2^ Rx). n=50	2.37 ± 0.31	0.044	2.280 – 2.457	1.78	3.42

n= total number examined, SD= Standard deviation, SE= Standard error of mean. **TB-Group (not on Rx)** =smear positive new cases not on treatment, **C-Group (non-TB)** = non TB control**. F-Group (2/12Rx** = patients that have completed 2 months of intensive treatment. **Rx=treatment**.

**Table 2:** relationship in mean serum sodium (mmol/l) in the 3 groups of study population

Sample groups		Mean difference	Standard error	p-values	95% Confidence interval	
					Lower bound	Upper bound
**TB-Group**	C- Group	-4.840	0.687	<0.001	-6.50	-3.18
	F- Group	-1.860	0.687	0.023	-3.52	-20
**C-Group**	TB-Group	4.840	0.687	<0.001	3.18	6.50
	F-Group	2.980	0.687	<0.001	1.32	4.64
**F-Group**	TB Group	1.860	0.687	0.023	0.20	3.52
	C- Group	-2.980	0.687	<0.001	-4.64	-1.32

**TB-Group (not on Rx) =** smear positive new cases not on treatment, **C-Group (non-TB) =** non TB control. **F-Group (2/12Rx =** patients that have completed 2 months of intensive treatment 50 from 2 months after initiation of treatment

Unlike serum sodium, the mean values of serum potassium did not vary significantly among the three groups (p=0.056, df=2, mean square=147, mean square=2.455) (between groups, F=2.945). However, post-hoc analysis showed significance difference in mean values of serum potassium in group C compared to F group (p<0.001), TB compared to F group (p<0.001), and TB compared to C group (p<0.001) (Table 3).

**Table 3:** post-hoc analysis of relationships of mean serum potassium in the 3 groups of the study population

Group	Group	Mean difference	SE	P-value	95% Confidence interval	
					Lower bound	Upper bound
**TB-Group**	C- Group	0.19100	0.18260	0.892	0.2512	0.6332
	F- Group	0.44180	0.18260	0.050	0.0004	0.8840
**C- Group**	TB Group	0.19100	0.18260	0.892	0.6332	0.2512
	F- Group	0.25080	0.18260	0.515	0.1914	0.6930
**F-Group**	TB- Group	0.44180	0.18260	0.050	0.8840	0.0004
	C- Group	0.25080	0.18260	0.515	0.6930	0.1914

Dependent variable: potassium (mmol/l). SE= Standard error. N= total number examined, SD= Standard deviation, SE= Standard error of mean. TB-Group (not on Rx) = smear positive new cases not on treatment, C-Group (non-TB) = non TB control. F-Group (2/12Rx = patients that have completed 2 months of intensive treatment50 from 2 months after initiation of treatment.

Among the three groups, the mean serum albumin level was highest in C group (38.31±2.01g/dl), followed by F group (36.77±3.85g/dl) and then TB group (31.51±4.34g/dl). The differences in mean values of serum albumin among the three groups was statistically significant, p<0.001, mean square between groups=635.522, mean square within groups=12.603, F=50.428. Post hoc analysis showed significant difference in the serum albumin levels both between and within the groups (Table 4).

**Table 4:** multivariate post-hoc analysis of relationships of mean serum albumin levels in the 3 groups of the study population

Group	Group	Mean difference	SE	P-value	95% Confidence interval	
					Lower bound	Upper bound
**TB-Group**	C- Group	-6.79820	71000	<0.001	-8.5176	-5.0788
	F- Group	-5.26180	71000	<0.001	-6.9812	-3.5424
**C- Group**	TB Group	6.79820	71000	<0.001	5.0788	8.5176
	F- Group	1.53640	71000	0.096	-.1830	3.2558
**F-Group**	TB- Group	5.26180	71000	<0.001	3.5424	6.9812
	C- Group	-1.53640	71000	0.096	-3.2558	.1830

Dependent variable: Albumin (g/dl). Bonferroni MD= mean difference, SE=Standard error. TB-Group (not on Rx) = smear positive new cases not on treatment, C-Group (non-TB) = non TB control. F-Group (2/12Rx = patients that have completed 2 months of intensive treatment 50 from 2 months after initiation of treatment.

The mean calcium level was highest for the F group (2.36±0.30mmol/l), followed by the C group (2.35±0.20mmol/l) and then the TB group (2.16±0.29mmol/l). The mean differences among the three groups were statistically significant (p<0.001), mean square between groups=0.611, mean square within the groups=0.074, F=8.286. Bonferroni between groups post-hoc analysis showed significance between groups C and F group (p=0.003), TB and F group (p=0.003), TB and C group (p=0.001) (Table 5).

**Table 5:** bivariate post-hoc analysis (Bonferri) of relationships between serum calcium and other serum biochemical variables in the 3 groups in study population

Sample group	Sample group	MD	SE	P-value	95% Confidence interval	
					Lower bound	Upper bound
**TB-Group**	C- Group	-0.18240	0.05431	0.003	-0.3139	-0.0509
	F- Group	-0.19940	0.05431	0.001	-0.3309	-0.0679
**C- Group**	TB Group	0.18240	0.05431	0.003	0.0509	0.3139
	F- Group	-0.01700	0.05431	1.000	-0.1435	0.1145
**F-Group**	TB- Group	0.19940	0.05431	0.001	0.0679	0.3309
	C- Group	0.01700	0.05431	1.000	-0.1145	0.1485

**Dependent variable: Calcium (mmol/l) Bonferroni MD**= mean difference, SE= Standard error. TB-Group (not on Rx) = smear positive new cases not on treatment, C-Group (non-TB) = non TB control. F-Group (2/12Rx = patients that have completed 2 months of intensive treatment 50 from 2 months after initiation of treatment

There was significant positive correlation between serum albumin and serum calcium (r=0.420, p<0.001) as well as serum sodium (r=0.310, p<0.001) in the study population (N=150). Similarly, the correlation between serum calcium and serum sodium was positive and significant (r=0.200, p=0.014). In contrast, the correlation between serum potassium and serum albumin and that between serum potassium and serum calcium was not significant (Table 6).

**Table 6:** correlation between variables in study population

		Potassium (mmol/l)	Albumin (g/dl)	Calcium (mmol/l)	Corrected calcium (mmol/l)	Sodium (mmol/l)
**Potassium (mmol/l)**	Pearson correlation	1	-122	0.049	0.048	0.041
	Sig (2-tailed)		0.136	0.551	0.559	0.618
	N	150	150	150	150	150
**Albumin**	Pearson correlation	-122	1	0.420	0.420	
	Sig (2-tailed)	0.136		0.000	<0.001	<0.001
	N	150	150	150	150	150
**Calcium (mmol/l)**	Pearson correlation	0.049	0.420	1	1.000	0.200
	Sig (2-tailed)	0.551	<0.001		<0.001	0.014
	N	150	150	150	150	150
**Corrected Calcium (mmol/L)**	Pearson correlation	0.048	0.420		1	0.201
	Sig (2-tailed)	0.559	<0.001	<0.001		0.014
	N	150	150	150	150	150
**Sodium (mmol/l)**	Pearson correlation	0.041	0.319	0.200	0.201	1
	Sig (2-tailed)	0.618	0.000	0.014	0.014	
	N	150	150	150	150	150

Correlation is significant at 0.01 level (2-tailed). Correlation is significant at the 0.05 level (2-tailed).

## Discussion

The study showed that biochemical changes in serum sodium, serum potassium, serum calcium and serum albumin were observed in TB infection and with TB treatment. The subgroup of patients with new case TB had significantly lower serum sodium than the control group and those on treatment. Both overt hyponatremia and low serum sodium levels in TB may be caused by any of these mechanisms: local invasion to the adrenals, hypothalamus, and pituitary gland or by syndrome of inappropriate anti-diuretic hormone (ADH) secretion if there is TB meningitis. This is similar to the finding of Nematollah Jonaidi Jafari *et al*. [4] after they reviewed 200 cases of TB which demonstrated lower serum sodium levels than those on treatment and controls. However, this is in contrast with a study by Olalekan *et al*. [5] in Southwestern Nigeria on electrolyte imbalance among TB patients on drugs, where serum sodium was significantly lower among those on treatment than the new cases not yet on treatment. The contrast may be due to the fact that in their study, HIV patients were included and no mention of their exclusion of kidney disease patients or other co-morbidities that may affect serum sodium. Difference in study population size may have also affected their results.

This study also showed significantly higher serum potassium in TB patients than control and those on treatment, in contrast to the findings of Olalekan *et al*. [5] in Southwestern Nigeria and Bhagyamma *et al*. [6] in India where patients with new TB infection had lower serum levels of potassium. *Mycobacterium tuberculosis* has a unique transcriptional response to changes in potassium levels both intra-mycobacteria and extra-mycobacteria [11]. Tuberculosis as an infection is a hypercatabolic state that is associated with cell and tissue destruction with movement of potassium into the serum. A study by Salina *et al*. [12] showed that potassium-deficient solid media retarded the growth of *M. tuberculosis* while potassium rich solid media and liquid media facilitated the growth of *M. tuberculosis* supporting the fact that mycobacteria may require a potassium-rich environment to proliferate.

There was also significantly low serum potassium among patients on follow-up treatment compared to cases and non-TB control group. Some anti-TB agents have been observed to cause hypokalaemia [9], and also vomiting being the major side effect of anti-TB drugs may lead to loss of potassium. Serum calcium was significantly lower in patients with TB than in the follow-up and the non-TB control group, similar to the findings of Ali-Gombe *et al*. [7] in Maiduguri and that of Rohini *et al*. [13] in India. Tuberculosis is a granulomatous disease which is associated with changes in calcium metabolism, inclusive of hypercalcaemia or hypocalcaemia. Hypocalcaemia in pulmonary TB was attributed to malnutrition, impaired absorption and Vitamin D deficiency.

The follow-up TB group on treatment had significantly higher levels of serum calcium than the new cases that were not yet on treatment and the non-TB control group. Furthermore, it is also similar to the findings of Ali-Gombe *et al*. [7]. The non-TB control group was composed mostly of civil servants working in the hospital environment who might have less exposure to sunlight and Vitamin D; and this may explain lower levels of calcium in them. This is in contrast with the study by Menon *et al*. [8] in Pakistan which reported higher serum calcium levels in TB group than the non-TB control group. Serum albumin was also noted to be significantly lower in TB new cases not yet on treatment than the non-TB control group. This is similar to the study by Ramakrishnan *et al*. [14] which documented low serum albumin in the TB patients when compared to the non-TB control group.

## Conclusion

Tuberculosis with or without anti-tuberculous medications was associated with significant reduction in serum albumin, serum sodium and serum calcium in this study. There is a need for clinicians to search for abnormalities of serum sodium, serum potassium, serum calcium and serum albumin in tuberculosis patients in routine clinical practice.

### What is known about this topic

- In tuberculosis patients, biochemical profiles are not routinely evaluated;
- Serum sodium has been found to be low in some tuberculosis patients, especially as a part of SIADH in pulmonary and meningeal tuberculosis;
- In tuberculosis infection, calcium metabolism has been reported to be affected.

### What this study adds

- This study shows significant reduction in serum albumin in tuberculosis patients;
- Serum calcium was shown to be significantly reduced in these patients;
- Significant reduction in serum sodium was also demonstrated in this study.
